# Association of rs7903146 (IVS3C/T) and rs290487 (IVS3C/T) Polymorphisms in *TCF7L2* with Type 2 Diabetes in 9,619 Han Chinese Population

**DOI:** 10.1371/journal.pone.0059053

**Published:** 2013-03-25

**Authors:** Jinjin Wang, Linlin Li, Jiatong Zhang, Jing Xie, Xinping Luo, Dahai Yu, Jingzhi Zhao, Tianping Feng, Chao Pang, Lei Yin, Fulan Hu, Jianfeng Zhang, Yan Wang, Qian Wang, Yujia Zhai, Haifei You, Tian Zhu, Dongsheng Hu

**Affiliations:** 1 Department of Epidemiology, College of Public Health, Zhengzhou University, Zhengzhou, Henan, People’s Republic of China; 2 Shenzhen University School of Medicine, Shenzhen, Guangdong, People’s Republic of China; 3 Department of Clinical Medicine, Zhengzhou University, Zhengzhou, Henan, People’s Republic of China; 4 Affiliated Hospital of Henan Military Region, Zhengzhou University, Zhengzhou, Henan, People’s Republic of China; 5 Department of Epidemiology, Public Health College, Harbin Medical University, Harbin, Heilongjiang, People’s Republic of China; 6 Henan Armed Police Corps Hospital, Zhengzhou, Henan, People’s Republic of China; Sapienza University, Italy

## Abstract

**Aims:**

We aimed to replicate the association of the rs290487 (IVS3C/T) and rs7903146 (IVS3C/T) polymorphisms of transcription factor 7-like 2 (*TCF7L2*) and type 2 diabetes mellitus (T2DM) in Han Chinese people in Henan province, China.

**Methods:**

In all, 1,842 patients with T2DM and 7,777 normal glucose-tolerant controls underwent genotyping for the T2DM-associated variants rs7903146 (IVS3C/T) and rs290487 (IVS3C/T). W performed a meta-analysis of the association of the risk alleles of rs7903146 (IVS3C/T) and rs290487 (IVS3C/T) in *TCF7L2* and T2DM in Han Chinese by combining previous studies with the present study.

**Results:**

We found that T2DM was associated with the CC genotype (1.364, 1.137–1.636, *p*  = 0.001), the recessive model (1.457, 1.156–1.838, *p*  = 0.001) of rs290487 (IVS3C/T) and haplotype CC (1.116, 1.034–1.204, *p*  = 0.004) in Han Chinese. Moreover, our meta-analyses supported the association of the T allele (IVS3C/T) of rs7903146 (1.36, 1.24–1.48; *p*  = 6.404×10^−12^) and T2DM but not the C allele of rs290487 (IVS3C/T) (0.99, 0.85–1.15, *p*  = 0.890) in Han Chinese. We found no interactions between behavioral risk factors (smoking, alcohol drinking, and physical activity) and rs7903146 (IVS3C/T) and rs290487 (IVS3C/T) polymorphisms.

**Conclusions:**

The CC genotype and the recessive model of the variant rs290487 (IVS3C/T) and CC haplotype of rs7903146 (IVS3C/T) and rs290487 (IVS3C/T) in *TCF7L2* may be associated with T2DM in Han Chinese people in Henan province, China.

## Introduction

Type 2 diabetes mellitus (T2DM) is a group of metabolic diseases characterized by hyperglycemia resulting from a progressive insulin secretory defect with insulin resistance [1]. The disease is now a pandemic and shows no signs of abatement especially in China. In 2011, the International Diabetes Federation estimated that 90 million people have diabetes in China; by 2030, this number will increase to 129.7 million people [2]. Diabetes and its complications have great economic impact on individuals, families, and health systems in China. From 2006 to 2015, China will spend 558 billion US dollars on heart disease, stroke and diabetes alone, according to World Health Organization estimates [3].

The mechanisms of T2DM remain unclear, but genetic susceptibility plays a crucial role in the etiology and manifestation of the disease [4], [5]. Transcriptionfactor7-like2 (*TCF7L2*) spans 215.9 kb on human chromosome 10q25 [6]with replicated linkage to T2DM in Mexican Americans [7]. The polymorphisms in *TCF7L2* gene have been evaluated in epidemiology studies of Chinese people and include rs7903146, rs290487, rs12255372, rs11196218, rs11196205, rs12775879, and DG10S478. The variants rs7903146 and rs290487 have shown inconsistent results in Han Chinese people. These two single-nucleotide polymorphisms (SNPs) have been most studied in Chinese people, but their association with T2DM in Han Chinese people is still controversial [8], [9], [10], [11], [12], [13], [14], [15], [16], [17], [18], [19], [20], [21], [22], [23], [24], [25], [26], [27]. Because of the low allele frequency of rs7903146 (IVS3C/T), previous studies were underpowered to detect its association with T2DM in Han Chinese. For rs290487 (IVS3C/T), the research has been mainly in the south of China, and data from northern China, especially in Henan province, are lacking.

Here, we aimd to investigate rs7903146 (IVS3C/T) and rs290487 (IVS3C/T) polymorphisms in *TCF7L2* in a case-control study of a large population of Han Chinese people to confirm the association of these 2 polymorphisms in *TCF7L2* and T2DM in Han Chinese people. We also performed a meta-analysis by combining these results with previous findings.

## Methods

### Study Population

All participants were of Northern Han Chinese ancestry and were recruited among local inhabitants of Henan Province from the outpatient clinics of several hospitals. T2DM was defined as fasting blood glucose ≧ 7.00 mmol/L or 2 h plasma glucose ≧ 11.0 mmol/L during an oral glucose tolerance test with diabetes clinical symptoms. We excluded patients with type 1 diabetes and other abnormal glucose tolerance and included patients with a previous diagnosis of T2DM in accordance with 2005 American Diabetes Association criteria [28]. Non-diabetic controls were from communities in Henan Province in 2008. Patients were between 20 and 85 years old. We excluded participants with body mass index (BMI) <18.50 and who were pregnant, handicapped, mentally disturbed, obese (caused by disease) or taking certain drugs, and had cancer. This study was approved by the Ethics Committees of Zhengzhou University, and informed consent was obtained from each participant before data collection. Demographic and anthropometric characteristics were collected by interviewer-administered questionnaire. Anthropometric data were body weight, body height, waist circumference, and blood pressure. An electronic sphygmomanometer was used to measure blood pressure.

### Biochemical Measurements

All blood samples were incubated with disodium EDTA for measuring, glucose level and non-EDTA for measuring total cholesterol (TC), triglycerides (TGs), and high- -density lipoprotein-cholesterol (HDL-C) by use automatic biochemical analysis, Low-density lipoprotein-cholesterol (LDL-C) level was calculated by the Freidwald formula [29].

### DNA Isolation and Genotyping

**Figure 1:**
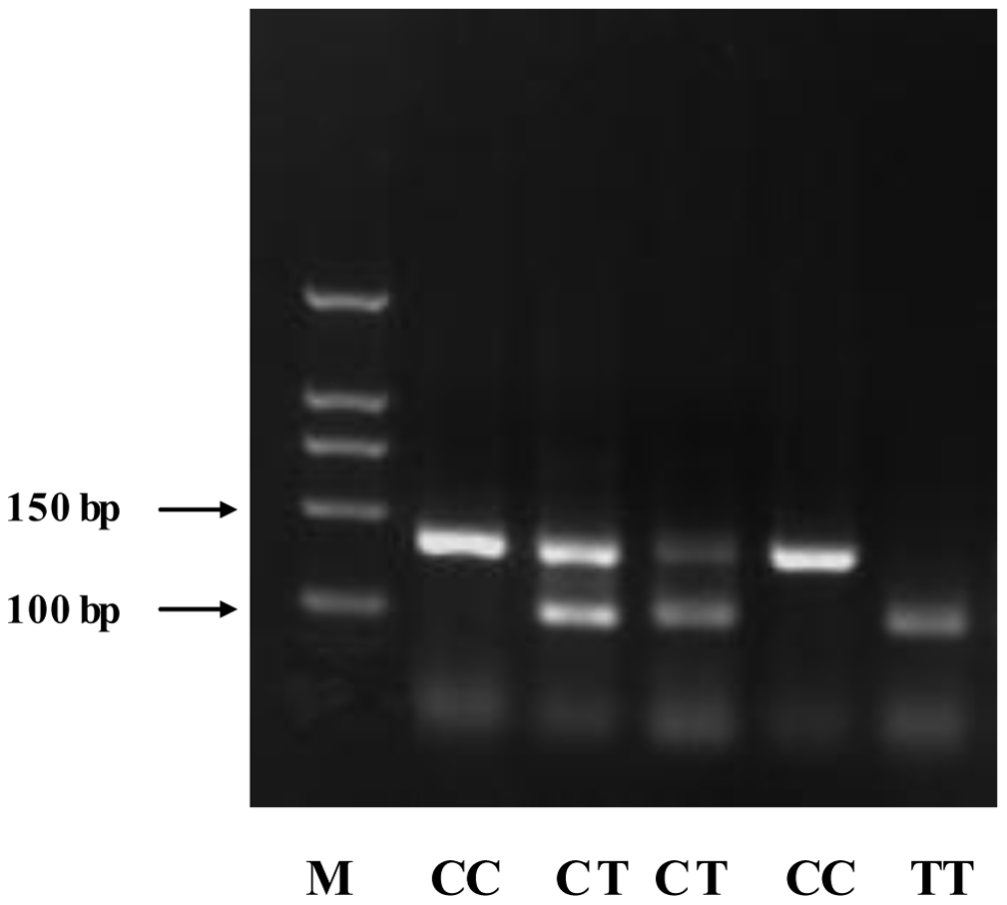
The image of genotyping result for rs7903146. Line 1: Maker; Line 2: CC genotype-139 bp; Line 3: CT genotype-139/111 bp; Line4: CT genotype-139/111 bp; Line 5: CC genotype-139 bp; Line 6: TT genotype-111 bp.

**Figure 2:**
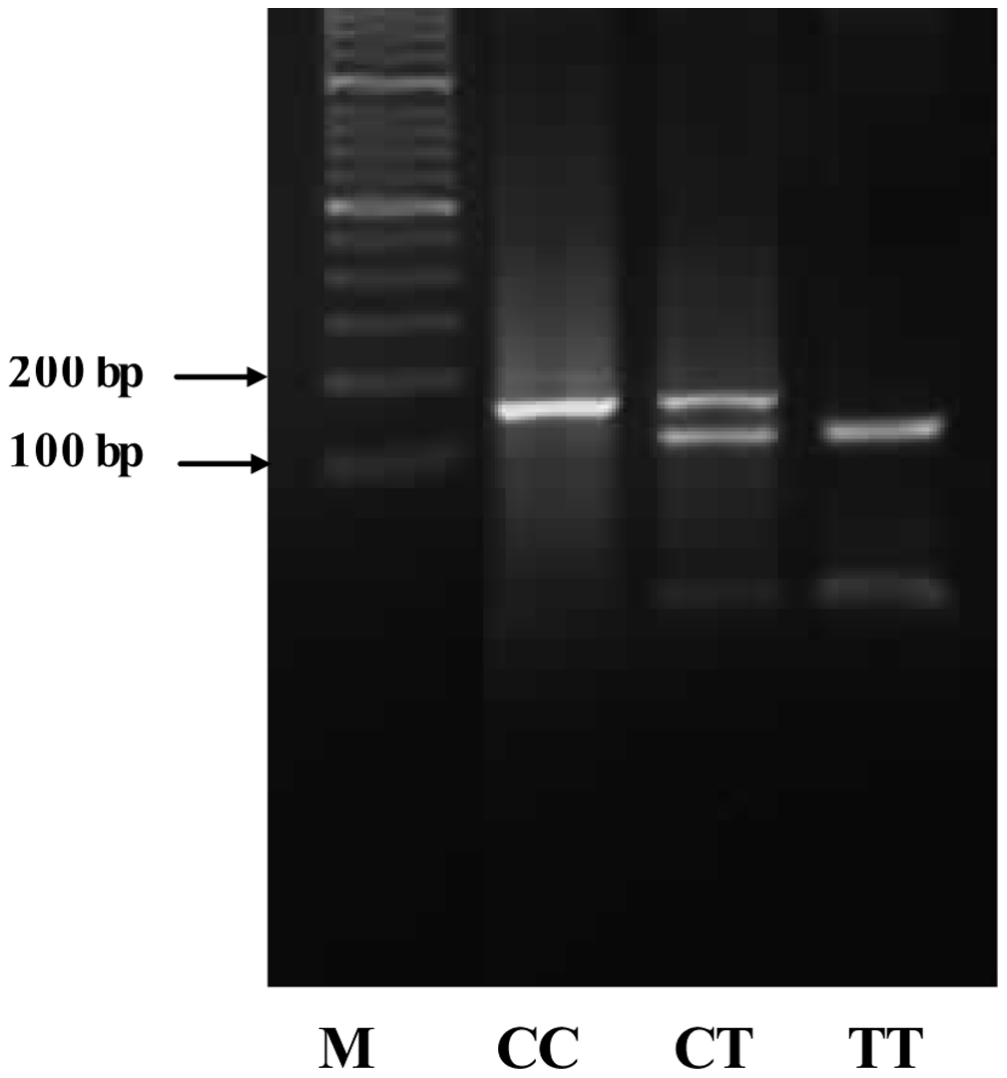
The image of genotyping result for rs290487. Line 1: Maker; Line 2: CC genotype-153 bp; Line 3: CT genotype-153/128 bp; Line4: TT genotype-128 bp.

Genomic DNA was extracted from whole blood by use of a blood genome DNA extraction kit (Yaneng BIO). Genotyping involved PCR- restriction fragment length polymorphism (PCR-RFLP). Primers were designed by use of OLIGO Primer Analysis Software 7.0 (Molecular Biology Insights, USA). The primer sequences and conditions for RFLP are in supplementary Table S1.The PCR samples were analyzed in a 20-µL reaction volume of 50 ng genomic DNA, 5 pmol each primer, 10 µL 2×Taq PCR mix (Laifeng BIO, containing 1 mmol/L MgCl_2_, 100 µmmol/L deoxynucleotide triphosphate (dNTP) and 0.5 U Taq polymerase), and the cycling conditions were 95°C for 10 min followed by 35 cycles of 95°C for 30 sec, 65°C [for rs7903146 (IVS3C/T)]/55°C [for rs290487 (IVS3C/T)] for 30 sec, and 72°C for 30 sec, and a final extension of 10 min at 72°C. PCR products were incubated for 10 hr with 3 U restriction enzyme in a 20-µL reaction volume and separated by 4% agarose gel electrophoresis. Genotyping success rates were 99.99% for rs7903146 (IVS3C/T) and rs290487 (IVS3C/T). To verify the reproducibility, we repeated 2% of samples at random as a quality controls for genotyping, and the concordance rate was 100% (see Fig. 1 and Fig. 2).

### Statistical Analysis

Categorical variables are described with number (percentage) and analyzed by chi-square test. Continuous variables are described with median (range) for non-normally data. Mann-Whitney-Wilcoxon and Kruskal-Wallis rank tests were used to assess differences between cases and controls. Hardy-Weinberg equilibrium for each SNP was separately calculated for cases and controls by use of the Fisher exact method [30]. Odds ratios (ORs), 95% confidence intervals (95% CIs) and corresponding *p* values for risk of T2DM were calculated by logistic regression analysis after adjusting for sex, age, anthropometric measurements (such as BMI, waist circumference, and blood pressure), and biochemical indexes such as TC, TG, HDL-C and LDL-C levels. The interactions between behavioral risk factors and SNPs were analyzed by adopting multiplicative interaction terms in a multivariate logistic regression model, including SNPs, risk factors, and interaction terms after adjusting for gender, age, and BMI. All tests were two sided and were considered statistically significant at *P*≤0.05. Haplotype analysis and linkage disequilibrium coefficients were calculated by use of Haploview 4.2 http://www.broad.mit.edu/haploview. *P*<0.05 was considered statistically significant. Calculation of population-attributable risk proportion (PARP) was based on the estimated ORs and genotypic frequencies of the SNP showing significant association with T2DM. Statistical analysis involved use of SPSS v17.0 for Windows (SPSS Inc., Chicago, IL). Power calculation involved use of PGA software.

### Meta-analysis of the Association of Risk Alleles of rs7903146 (IVS3C/T) and rs290487 (IVS3C/T) of *TCF7L2* and T2DM in Han Chinese

We searched MEDLINE via PubMed, EMBASE, Cohorane, and Chinese databases (CNKI, CQVIP, and Wanfang Databases) from January 2007 to February 2012 using the keywords: ‘*TCF7L2*’, ‘genetic polymorphism’, ‘Chinese’ and ‘T2D/T2DM’ for articles related to Chinese participants published in English and Chinese. We used the Metan module within the STATA 11.0 software for the meta-analysis of allele frequencies by combining the previous published data with our data. The strength of association of risk alleles and T2DM was measured by ORs with 95% CIs. The Z test was used to determine the statistical significance of the pooled OR. Power calculation involved use of PGA software. Calculation of Population-attributable risk proportion (PARP) was based on the estimated ORs and risk allele frequencies of the SNP showing significant association with T2DM.

## Results

### Characteristic of the Study Participants

We included 1,842 T2DM patients (925 males) and 7,777 controls (3,214 males) (see supplementary Table S2). The 2 groups differed in age: 54 (range 20–85) years for T2DM patients, and 49 (range 25–75) years for controls. Compared with controls, diabetic patients had significantly higher anthropometric and metabolic measurements (*p*<0.001).

### Association of rs290487 (IVS3C/T) and rs7903146 (IVS3C/T) Polymorphisms with T2DM

The distributions of both risk alleles in these two SNPs differed between cases and controls (see supplementary Table S3). Genotypes of rs290487 (IVS3C/T) (*p*  = 7.038×10^−11^) but not rs7903146 (IVS3C/T) (*p*  = 0.063) were associated with T2DM (see supplementary Table S4). For controls, genotype distributions for SNPs in *TCF7L2* were all in Hardy-Weinberg equilibrium (*p*>0.05).

The CT genotype (0.838, 0.750–0.936, *p*  = 0.013) and CC versus TT genotype (1.403, 1.207–1.631, *p*  = 1.077×10^−5^) and the recessive model (OR 1.371, 95% CI, 1.177–1.596, *p*  = 0.004) of rs290487 (IVS3C/T) were associated with T2DM. After adjustmet for potential confounders such as sex, age, anthropometric measurements, and metabolic measurements, the CC genotype (1.364, 1.137–1.636, *p*  = 0.001) and the recessive model (1.457, 1.156–1.838, *p*  = 0.001) and were associated with T2DM. The PARP was 4.05% for the CC genotype of rs290487 (IVS3C/T). No genotypes for rs7903146 (IVS3C/T) was associated with T2DM in the Han Chinese population with adjusted or unadjusted analysis.

### Haplotype Analyses

We found 4 haplotypes involving rs7903146 (IVS3C/T) and rs290487 (IVS3C/T) in *TCF7L2* in study participants. The D’ value for rs7903146 (IVS3C/T) and rs290487 (IVS3C/T) was 0.028 (r^2^ = 0.001), which indicated weak linkage disequilibrium for the SNPs. After analyzing all estimated haplotypes, haplotype CC was associated with T2DM (OR 1.116, 95% CI 1.034–1.204, *p*  = 0.004, see supplementary Table S5).

### Interactions between Behavioral Risk Factors and SNPs

Because multivariate regression analysis revealed a significant association of behavioral risk factors (included smoking, alcohol drinking, and physical activity) and T2DM, we analyzed the interactions of behavioral risk factors and SNPs in *TCF7L2* but found no interactions after adjustment for gender, age, and BMI (*p*>0.05) (see supplementary Table S6).

### Clinical and Metabolic Characteristics of Controls by rs7903146 (IVS3C/T) and rs290487 (IVS3C/T) Genotypes

Linear regression analysis revealed no association of clinical and metabolic characteristics of controls and the distribution of studied genotypes with adjustment for age, gender and BMI (see supplementary Table S7).

### Meta-analysis of Association of Risk Alleles of rs7903146 (IVS3C/T) and rs290487 (IVS3C/T) of *TCF7L2* and T2DM in Han Chinese

Our meta-analysis included 9 studies comprising 5,215 cases and 10,772 controls for rs290487 (IVS3C/T) and 15 studies comprising 9,744 cases and 15,246 controls for rs7903146 (IVS3C/T) in Han Chinese [8]–[27] (see supplementary Table S8). The meta-analysis revealed a significant association of the T allele of rs7903146 (IVS3C/T) in *TCF7L2* and T2DM (OR 1.23, 95% CI 1.13 to 1.33, *p*<0.001) but not the C allele of rs290487 (IVS3C/T) (0.99, 0.85–1.15; *p*  = 0.890). For the association of the T allele of rs7903146 (IVS3C/T) and T2DM, the PARP was 1.77% and the power of the meta-analysis was 100%.

## Discussion

Our findings revealed an association of T2DM and the risk allele C and CC genotype of the SNP rs290487 (IVS3C/T) of *TCF7L2* and the haplotype CC in Han Chinese in Henan province in China. We found no association of clinical and metabolic characteristics and the distribution of the 2 SNPs in controls. As well, we found no interactions between behavioral risk factors and distribution of the SNPs in Han Chinese in Henan province, China.

We found an association of the CC genotype (adjusted OR 1.364, 1.137–1.636, *p*  = 0.001) and the recessive model (adjusted OR 1.457, 1.156–1.838, *p*  = 0.001) of rs290487 (IVS3C/T) and T2DM, and the power of our study was 100% to detect association based on the prevalence of T2DM in China is 9.7%. This founding is familiar to our previous meta-analysis findings for southern China (OR 1.54, 1.22–1.94, *I^2^* = 0.00%, *p*<0.001) and studies by H. Zhu et al. (1.73, 1.04–2.87) [27] and Ren et al. (1.73, 1.00–2.25) [8]. However, results from other studies were opposite, such as the results of YC. Chan et. al [9], M. Yu et. al [13], and Q. Huang et. al [23]. The main reason might due to the complex of the composition of the Chinese population and the great differences among ethnic groups in genetics and living environment.

We found no association of any genotypes for rs7903146 (IVS3C/T) and T2DM. This founding was similar to that form almost all studies performed in China, except for Z. Wang et al [18] and X.Tang et al study [26] in Chongqing and Chengdu. China is divided into north and south section with the Yangtze River as the approximate boundary, and Chongqing and Chengdu belong to southern China, whereas Henan belongs to northern China. The environment, diet, and lifestyles greatly differ which might explain the inconsistent results. Because of the low frequency of the risk allele T in rs7903146 (IVS3C/T) in the NCBI database, the sample sizes are important for detecting associations.

Both our previous meta-analysis and present meta-analysis indicated the strong association of rs7903146 (IVS3C/T) and T2DM, with the power of the meta-analysis is 100% for the risk allele of rs7903146 (IVS3C/T). This result is inconsistent with our study findings. The reasons for this discrepancy may be because first, the meta-analysis pooled all results from China, and the Han Chinese are not a genetically homogenous group, so regional diversity may contribute to the discrepancy. Second, because of the lack of full information about some potential confounders such as gender, age, and BMI of subjects included in the meta-analysis, we could not perform a stratified or interaction analysis based on these factors. However, in our study, we attempted to adjust findings for the potential confounders.

In the Bodhini. D et al [31] and Lehman et al studies [32], rs7903146 (IVS3C/T) variants were associated with increased 2-hr plasma glucose levels in Asian Indians and Caucasians, respectively, and in Liu PH et al study [33], the C allele of rs290487 (IVS3C/T) was associated with 60-, 90-, and 120-min glucose concentrations#. However, because we lacked this information, we did not analyze these indexes. We assessed other clinical and metabolic characteristics of normal glucose-tolerant participants and found no associations between the indexes and the 2 SNPs.

Henan is the most populated province in China, but data were lacking on the association of rs7903146 (IVS3C/T) and rs290487 (IVS3C/T) polymorphisms and T2DM. Our study is the first to assess the associations with SNPs in Han Chinese in Henan province. As well, we analyzed the associations of metabolic measurements and genotypes of the 2 loci in Han Chinese population for the first time. In addition, our meta-analysis, by combining our data and published data, provides strong evidence for the association of the risk alleles of these 2 SNPs and T2DM in Han Chinese. Finally, the power of our study to detect the association of rs290487 (IVS3C/T) and T2DM in our study population was 100% by PGA.

In conclusion, our findings suggest that the genotypes of rs7903146 (IVS3C/T) in *TCF7L2* have no major impact on T2DM; however, the CC genotype and the recessive model of rs290487 (IVS3C/T) and the haplotype CC of rs7903146 (IVS3C/T) and rs290487 (IVS3C/T) in *TCF7L2* gene are associated with T2DM. We found no association of behavioral risk factors and distribution of rs7903146 (IVS3C/T) and rs290487 (IVS3C/T) in Han Chinese in Henan province, China. However, more representative and comprehensive studies of people with different ethnic backgrounds are needed to clarify the mechanisms and underlying genetic effects of T2DM in the Chinese population.

## Supporting Information

Table S1 **Primer sequences and restriction enzymes.**
(DOC)

Table S2 **Characteristics of study participants.**
(DOC)

Table S3 **Genotypic and allelic distributions of single nucleotide polymorphisms in** ***TCF7L2*** **gene among Han Chinese in China.**
(DOC)

Table S4 **Association of single nucleotide polymorphisms in** ***TCF7L2*** **gene and type 2 diabetes mellitus in Han Chinese in China.**
(DOC)

Table S5 **Associations of haplotypes of SNPs in** ***TCF7L2*** **gene and T2DM.**
(DOC)

Table S6 **Interaction of behavioral factors and genotypes of single nucleotide polymorphisms (SNPs) in** ***TCF7L2*** **for type 2 diabetes mellitus.**
(DOC)

Table S7 **Association of clinical and biochemical characteristics of normal glucose-tolerant participants and genotype.**
(DOC)

Table S8 **Meta-analyses of risk alleles in** ***TCF7L2*** **and T2DM in Han Chinese population.**
(DOC)
