# Supplementary material for: Association of rs7903146 (IVS3C/T) and rs290487 (IVS3C/T) Polymorphisms in TCF7L2 with Type 2 Diabetes in 9,619 Han Chinese Population
Source: PLoS One. 2013 Mar 25;8(3):e59053. doi: 10.1371/journal.pone.0059053 (PMC3607568; doi:10.1371/journal.pone.0059053)
Supplement: Table S1 — Primer sequences and restriction enzymes. (DOC) [file pone.0059053.s001.doc]

**Table S1. Primer sequences and restriction enzymes**

| Polymorphisms | Primer sequences | Restriction enzymes (T) |
| --- | --- | --- |
| rs7903146 (IVS3C-T) | 5’-ACAATTAGAGAGCTAAGCACTTTTTAAATA-3’ (F) | SspI (Fermantas), (37℃) |
|  | 5’-CTAACCTTTTCCTAGTTATCTGACATTG-3’ (R) |
| rs290487 (IVS3C-T) | 5’-AGGAGGCTGCCATATTGTTTACTT-3’ (F) | AccII (Fermantas), (37℃) |
|  | 5’-ACACCTTTCTCATTTTCAATTTCGC-3’ (R) |
