# Supplementary material for: Association of rs7903146 (IVS3C/T) and rs290487 (IVS3C/T) Polymorphisms in TCF7L2 with Type 2 Diabetes in 9,619 Han Chinese Population
Source: PLoS One. 2013 Mar 25;8(3):e59053. doi: 10.1371/journal.pone.0059053 (PMC3607568; doi:10.1371/journal.pone.0059053)
Supplement: Table S2 — Characteristics of study participants. (DOC) [file pone.0059053.s002.doc]

**Table S2. Characteristics of study participant**s

| Characteristics | Cases  (n = 1,842) | Controls  (n = 7,777) | *P* |
| --- | --- | --- | --- |
| Sex |  |  |  |
| Male | 925 (50.20) | 3,214 (41.32) | < 0.001 |
| Female | 917 (49.80) | 4,563 (58.68) |
| Age (years) | 54 (20-85) | 49 (25-75) | < 0.001 |
| Body mass index (kg/m^2^) | 27.38 (18.51-50.45) | 23.83 (18.51-43.50) | < 0.001 |
| Waist circumference (cm) | 90.50 (60.60-200.00) | 81.35 (58.00-155.00) | < 0.001 |
| SBP (mmHg) | 128.00 (87.67-218.67) | 121.00 (81.33-218.33) | < 0.001 |
| DBP (mmHg) | 81.33 (44.00-136.00) | 77.00 (49.00-140.00) | < 0.001 |
| Fasting plasma glucose (mmol/L) | 7.63 (3.45-27.13) | 5.19 (3.23-6.09) | < 0.001 |
| HDL-C (mmol/L) | 1.10 (0.55-4.73) | 1.14 (0.47-2.48) | < 0.001 |
| LDL-C (mmol/L) | 2.94 (0.20-10.47) | 2.40 (-1.70-8.80) | < 0.001 |
| TG (mmol/L) | 1.37 (0.38-14.28) | 1.32 (0.33-11.06) | < 0.001 |
| TC (mmol/L) | 4.86 (1.58-13.66) | 4.28 (1.66-10.23) | < 0.001 |

Data are number (%) or median (range).
