# Supplementary material for: Association of rs7903146 (IVS3C/T) and rs290487 (IVS3C/T) Polymorphisms in TCF7L2 with Type 2 Diabetes in 9,619 Han Chinese Population
Source: PLoS One. 2013 Mar 25;8(3):e59053. doi: 10.1371/journal.pone.0059053 (PMC3607568; doi:10.1371/journal.pone.0059053)
Supplement: Table S3 — Genotypic and allelic distributions of single nucleotide polymorphisms in TCF7L2 gene among Han Chinese in China. (DOC) [file pone.0059053.s003.doc]

**Table S3. Genotypic and allelic distributions of single nucleotide polymorphisms in *TCF7L2* gene among Han Chinese in China**

| SNPs | Cases | Controls | *P* |
| --- | --- | --- | --- |
| rs7903146(IVS3C-T) |  |  |  |
| TT | 6 (0.32) | 27 (0.35) | 0.063 |
| CT | 283 (15.36) | 1,032 (13.27) |
| CC | 1,553 (84.31) | 6,718 (86.38) |
| T | 295 (8.01) | 1,086 (6.98) | 0.030 |
| C | 3,389 (91.99) | 14,468 (93.02) |
| rs290487(IVS3C-T) |  |  |  |
| TT | 798 (43.32) | 3,276 (42.12) | 7.038×10^-11^ |
| CT | 734 (39.85) | 3,600 (46.29) |
| CC | 310 (16.83) | 901 (11.59) |
| T | 2,330 (63.25) | 10,152 (65.27) | 0.021 |
| C | 1,354 (36.75) | 5,402 (34.73) |

Data are number (%).
