# Supplementary material for: Association of rs7903146 (IVS3C/T) and rs290487 (IVS3C/T) Polymorphisms in TCF7L2 with Type 2 Diabetes in 9,619 Han Chinese Population
Source: PLoS One. 2013 Mar 25;8(3):e59053. doi: 10.1371/journal.pone.0059053 (PMC3607568; doi:10.1371/journal.pone.0059053)
Supplement: Table S4 — Association of single nucleotide polymorphisms in TCF7L2 gene and type 2 diabetes mellitus in Han Chinese in China. (DOC) [file pone.0059053.s004.doc]

**Table S4. Association of single nucleotide polymorphisms in *TCF7L2* gene and type 2 diabetes mellitus in Han Chinese in China**

| Genotypes | Unadjusted ORs (95%CI) | *P* | Adjusted ORs^*^ (95% CI) | *P^*^* |
| --- | --- | --- | --- | --- |
| rs7903146 (IVS3C-T) |  |  |  |  |
| CC | 1 |  | 1 |  |
| TC | 1.153 (1.000-1.331) | 0.051 | 1.086 (0.915-1.290) | 0.347 |
| TT | 0.856 (0.352-2.084) | 0.732 | 0.988 (0.350-1.290) | 0.982 |
| TT vs. CT+CC | 1.129 (0.843-1.514) | 0.723 | 1.246 (0.780-1.991) | 0.358 |
| TT+CT vs. CC | 1.026 (0.891-1.180) | 0.415 | 1.012 (0.810-1.264) | 0.917 |
| rs290487 (IVS3C-T) |  |  |  |  |
| TT | 1 | - | 1 | - |
| CT | 0.838 (0.750-0.936) | 0.013 | 0.851 (0.747-1.134) | 0.163 |
| CC | 1.403 (1.207-1.631) | 1.077×10^-5^ | 1.364 (1.137-1.636) | 0.001 |
| CC vs. CT+TT | 1.371 (1.177-1.596) | 0.004 | 1.457 (1.156-1.838) | 0.001 |
| CC+CT vs. TT | 0.932 (0.842-1.033) | 0.181 | 0.984 (0.841-1.152) | 0.843 |

*Adjusted for sex, age, anthropometric measurements, TC, TG, HDL-C, and LDL-C.
