# Supplementary material for: Association of rs7903146 (IVS3C/T) and rs290487 (IVS3C/T) Polymorphisms in TCF7L2 with Type 2 Diabetes in 9,619 Han Chinese Population
Source: PLoS One. 2013 Mar 25;8(3):e59053. doi: 10.1371/journal.pone.0059053 (PMC3607568; doi:10.1371/journal.pone.0059053)
Supplement: Table S5 — Associations of haplotypes of SNPs in TCF7L2 gene and T2DM. (DOC) [file pone.0059053.s005.doc]

**Table S5. Associations of haplotypes of SNPs in *TCF7L2* gene and T2DM**

|  | Haplotypes | | Cases | Controls | ORs (95%CI) | *P* |
| --- | --- | --- | --- | --- | --- | --- |
|  | rs7903146(IVS3C-T) | rs290487(IVS3C-T) |  |  |  |  |
| 1 | C | C | 1,258 (34.2) | 4,936 (31.7) | 1.116 (1.034-1.204) | 0.004 |
| 2 | C | T | 2,131 (57.8) | 9,532 (61.3) | 0.866 (0.805-1.932) | 0.067 |
| 3 | T | C | 96 (2.6) | 466 (3.0) | 0.862 (0.689-1.077) | 0.191 |
| 4 | T | T | 199 (5.4) | 620 (4.0) | 1.379 (0.971-1.625) | 0.231 |

Data are number (%).
