# Supplementary material for: Association of rs7903146 (IVS3C/T) and rs290487 (IVS3C/T) Polymorphisms in TCF7L2 with Type 2 Diabetes in 9,619 Han Chinese Population
Source: PLoS One. 2013 Mar 25;8(3):e59053. doi: 10.1371/journal.pone.0059053 (PMC3607568; doi:10.1371/journal.pone.0059053)
Supplement: Table S6 — Interaction of behavioral factors and genotypes of single nucleotide polymorphisms (SNPs) in TCF7L2 for type 2 diabetes mellitus. (DOC) [file pone.0059053.s006.doc]

**Table S6 Interaction of behavioral factors and genotypes of single nucleotide polymorphisms (SNPs) in *TCF7L2* for type 2 diabetes mellitus**

| SNP | Genotype | Behavioral risk factors | | | | | | | | | |
| --- | --- | --- | --- | --- | --- | --- | --- | --- | --- | --- | --- |
|  |  | Smoking status | | | Alcohol drinking status | | | | Physical Activity | | |
|  |  | GT × Never | GT × Current smoker | GT ×Ex-smoker | GT × Never | GT × Slight | GT × Moderate | GT × Severe | GT × Slight | GT × Moderate | GT × Severe |
| rs7903146 | CC | 1 | 1.053 (0.431-2.573) 0.910 | 0.140 (0.011-1.776) 0.129 | 1 | 0.490 (0.079-3.059) 0.446 | 1.465 (0.322-6.672) 0.622 | 0.613 (0.103-3.642) 0.613 | 1 | 0.261 (0.094-0.721) 0.010 | 0.208 (0.093-0.465) 1.13 × 10^-5^ |
|  | CT | 1.176 (0.632-2.187) 0.609 | 0.838 (0.318-2.208) 0.721 | 1.317 (0.093-18.650) 0.839 | 1.176 (0.632-2.187) 0.609 | 0.521 (0.070-3.891) 0.525 | 1.034 (0.174-6.151) 0.971 | 0.988 (0.143-6.836) 0.990 | 1.176 (0.632-2.187) 0.609 | 0.845 (0.273-2.616) 0.770 | 0.843 (0.352-2.018) 0.701 |
|  | TT | 1.149 (0.652-2.025) 0.631 | 0.744 (0.310-1.783) 0.507 | 2.334 (0.193-28.173) 0.505 | 1.149 (0.652-2.025) 0.631 | 0.875 (0.163-4.701) 0.877 | 1.387 (0.300-6.409) 0.675 | 1.103 (0.186-6.530) 0.914 | 1.149 (0.652-2.025) 0.631 | 1.124 (0.409-3.088) 0.820 | 0.863 (0.390-1.909) 0.716 |
| rs290487 | TT | 1 | 1.053 (0.431-2.573) 0.910 | 0.140 (0.011-1.776) 0.129 | 1 | 0.490 (0.079-3.059) 0.446 | 1.465 (0.322-6.672) 0.622 | 0.613 (0.103-3.642) 0.613 | 1 | 0.261 (0.094-0.721) 0.010 | 0.208 (0.093-0.465) 1.13 × 10^-5^ |
|  | TC | 0.994 (0.815-1.212) 0.951 | 0.777 (0.554-1.090) 0.144 | 1.034 (0.504-2.123) 0.927 | 0.994 (0.815-1.212) 0.951 | 2.112 (0.820-5.435) 0.121 | 0.591 (0.312-1.118) 0.106 | 0.752 (0.389-1.456) 0.398 | 0.994 (0.815-1.212) 0.951 | 0.790 (0.533-1.171) 0.241 | 0.990 (0.737-1.330) 0.947 |
|  | CC | 1.348 (0.979-1.858) 0.067 | 0.697 (0.411-1.183) 0.181 | 2.138 (0.782-5.846) 0.139 | 1.348 (0.979-1.858) 0.067 | 2.102 (0.569-7.759) 0.265 | 0.592 (0.215-1.628) 0.310 | 1.337 (0.471-3.795) 0.586 | 1.348 (0.979-1.858) 0.067 | 0.882 (0.494-1.576) 0.672 | 1.185 (0.763-1.842) 0.449 |

Data are OR (95% CI), *P* value

GT, genotype adjusted for sex, age, and body mass index

*P* value for testing effect of modification by behavior risk factors using an interaction term of status of behavior risk factors and genotypes of SNPs by multivariate logistic regression.
