# Supplementary material for: Association of rs7903146 (IVS3C/T) and rs290487 (IVS3C/T) Polymorphisms in TCF7L2 with Type 2 Diabetes in 9,619 Han Chinese Population
Source: PLoS One. 2013 Mar 25;8(3):e59053. doi: 10.1371/journal.pone.0059053 (PMC3607568; doi:10.1371/journal.pone.0059053)
Supplement: Table S7 — Association of clinical and biochemical characteristics of normal glucose-tolerant participants and genotype. (DOC) [file pone.0059053.s007.doc]

**Table S7. Association of clinical and biochemical characteristics of normal glucose-tolerant participants and genotype**

| Characteristics | rs7903146(IVS3C-T) | | | | rs290487(IVS3C-T) | | | |
| --- | --- | --- | --- | --- | --- | --- | --- | --- |
|  | CC | TC | TT | *P* | CC | CT | TT | *P* |
| Fasting plasma glucose (mmol/L) | 5.19 (3.23-6.09) | 5.19 (3.49-6.09) | 5.43 (4.45-6.06) | 0.944 | 5.20 (3.49-6.09) | 5.19 (3.23-6.09) | 5.20 (3.25-6.09) | 0.598 |
| TG (mmol/L) | 1.32 (0.33-11.06) | 1.32 (0.33-10.30) | 1.69 (0.50-4.02) | 0.882 | 1.34 (0.37-10.87) | 1.32 (0.33-11.06) | 1.32 (0.33-11.02) | 0.475 |
| TC (mmol/L) | 4.28 (1.66-10.23) | 4.24 (2.40-9.98) | 4.28 (3.01-5.86) | 0.694 | 4.28 (1.72-8.78) | 4.29 (1.66-9.98) | 4.26 (1.79-10.23) | 0.703 |
| HDL-C (mmol/L) | 1.14 (0.47-2.48) | 1.15 (0.46-2.30) | 1.12 (0.61-1.86) | 0.342 | 1.13 (0.46-2.13) | 1.14 (0.48-2.40) | 1.14 (0.47-2.48) | 0.534 |
| LDL-C (mmol/L) | 2.40 (0.05-8.80) | 2.40 (0.10-6.80) | 2.40 (1.40-3.70) | 0.915 | 2.40 (0.10-6.50) | 2.40 (0.05-8.80) | 2.50 (0.10-6.50) | 0.634 |
| SBP (mmHg) | 121.00 (83.00-218.00) | 121.00 (81.00-211.00) | 122.00 (100.00-162.00) | 0.243 | 121.00 (81.00-208.00) | 121.00 (84.00-218.00) | 121.00 (83.00-216.00) | 0.768 |
| DBP (mmHg) | 77.00 (49.00-140.00) | 77.00 (50.00-136.00) | 76.00 (64.00-93.00) | 0.899 | 77.00 (50.00-136.00) | 77.00 (49.00-140.00) | 77.00 (50.00-136.00) | 0.955 |

Data are median (range).
