# Supplementary material for: Association of rs7903146 (IVS3C/T) and rs290487 (IVS3C/T) Polymorphisms in TCF7L2 with Type 2 Diabetes in 9,619 Han Chinese Population
Source: PLoS One. 2013 Mar 25;8(3):e59053. doi: 10.1371/journal.pone.0059053 (PMC3607568; doi:10.1371/journal.pone.0059053)
Supplement: Table S8 — Meta-analyses of risk alleles in TCF7L2 and T2DM in Han Chinese population. (DOC) [file pone.0059053.s008.doc]

**Table S8. Meta-analyses of risk alleles in *TCF7L2* and T2DM in Han Chinese population**

| SNPs | Authors | Year | Regions | Risk / non-risk allele | RAF (case/control) | Allelic ORs (95% CI) | *P* |
| --- | --- | --- | --- | --- | --- | --- | --- |
| rs290487(IVS3C-T) | Chang Yicheng | 2007 | Taiwan | C/T | 0.42 / 0.36 | 1.32 (1.14, 1.53) |  |
|  | Zhang Ying | 2008 |  |  | 0.36 / 0.40 | 0.84 (0.70, 1.01) |  |
|  | Q.Ren | 2008 | Beijing |  | 0.39 / 0.35 | 1.18 (0.99, 1.42) |  |
|  | Zhang Yong | 2009 | Jinan |  | 0.39 / 0.36 | 1.16 (0.77, 1.74) |  |
|  | Zou Yulian | 2009 | Kunming |  | 0.35 / 0.30 | 1.26 (0.86, 1.83) |  |
|  | Yu Min | 2009 | Changsha |  | 0.37 / 0.38 | 0.95 (0.73, 1.24) |  |
|  | Zhu Hui | 2011 | Anhui |  | 0.41 / 0.34 | 0.57 (0.45, 0.72) |  |
|  | Qiao Huang | 2012 | Haerbin |  | 0.38 / 0.43 | 0.84 (0.72, 0.99) |  |
|  | Our study | 2012 | Henan |  | 0.37 / 0.35 | 1.09 (1.01, 1.18) |  |
|  | Combined all |  |  |  | 0.38 / 0.36 | 0.99 (0.85, 1.15) | 0.890 |
| rs7903146(IVS3C-T) | Zeng Qingcui | 2007 | Chongqing | T/C | 0.04 / 0.01 | 3.41 (0.74, 15.75) |  |
|  | Chang Yicheng | 2007 | Taiwan |  | 0.02 / 0.03 | 0.81 (0.52, 1.27) |  |
|  | Maggie C.Y.Ng | 2008 | Hong Kong |  | 0.03 / 0.02 | 1.31 (0.95, 1.80) |  |
|  | Q.Ren | 2008 | Beijing |  | 0.05 / 0.03 | 1.56 (0.97, 2.49) |  |
|  | Wang Zhihong | 2008 | Chongqing |  | 0.09 / 0.04 | 2.29 (1.45, 3.60) |  |
|  | Lou Qinglin | 2009 | Jiangsu |  | 0.07 / 0.02 | 1.61 (0.96, 2.62) |  |
|  | Tang Xin | 2009 | Chengdu |  | 0.06 / 0.04 | 1.53 (1.22, 1.94) |  |
|  | Zheng Xiaoya | 2010 | Chongqing |  | 0.06 / 0.04 | 1.66 (1.23, 2.24) |  |
|  | Zhang Lin | 2010 | Changsha |  | 0.05 / 0.04 | 1.36 (0.67, 2.73) |  |
|  | Jie Wen | 2010 | Shanghai |  | 0/05 / 0/04 | 1.41 (0.74, 2.68) |  |
|  | Chen Guanya | 2011 | Enshi |  | 0/15 / 0.09 | 1.81 (1.21, 2.71) |  |
|  | Zhu Hui | 2011 | Anhui |  | 0.03 / 0.02 | 1.37 (0.68, 2.76) |  |
|  | Zhao Ting | 2011 | Qingdao |  | 0.06 / 0.02 | 3.35 (1.05, 10.69) |  |
|  | Wang Yupin | 2011 | Yanbian |  | 0.02 / 0.00 | 10.07 (0.55, 183.20) |  |
|  | Our study | 2012 | Henan |  | 0.08 / 0.07 | 1.16 (1.01, 1.33) |  |
|  | Combined all |  |  |  | 0.06 / 0.05 | 1.36 (1.24, 1.48) | 6.404×10^-12^ |

RAF: risk allele frequency
